# Prevention and Control of Biofouling Coatings in *Limnoperna fortunei*: A Review of Research Progress and Strategies

**DOI:** 10.3390/polym16213070

**Published:** 2024-10-31

**Authors:** Hailong Zhang, Qingjie Ding, Yonghui Zhang, Guangyi Lu, Yangyu Liu, Yuping Tong

**Affiliations:** 1 School of Materials Science and Engineering, North China University of Water Resources and Electric Power, Zhengzhou 450045, China; liuyangyu@ncwu.edu.cn (Y.L.); yptong_zz@163.com (Y.T.); 2 Sinohydro Bureau 11 Co., Ltd., Zhengzhou 450000, China; 17737607882@189.cn (Q.D.); 16638066318@163.com (G.L.)

**Keywords:** antifouling coatings, *Limnoperna fortunei*, eco-friendly coatings, environmental sustainability

## Abstract

The increasing environmental concerns of conventional antifouling coatings have led to the exploration of novel and sustainable solutions to address the biofouling caused by *Limnoperna fortunei*. As a rapidly expanding invasive species, the fouling process of *Limnoperna fortunei* is closely associated with microbial fouling, posing significant threats to the integrity of aquatic infrastructure and biodiversity. This review discusses recent progress in the development of non-toxic, eco-friendly antifouling coatings that are designed to effectively resist biofouling without using toxic chemicals. Recent research has focused on developing novel non-toxic coatings that integrate natural bioactive components with advanced material technologies. These formulations not only meet current environmental standards and exhibit minimal ecological impact, but also possess significant potential in preventing the attachment, growth, and reproduction of *Limnoperna fortunei*. This review aims to provide scientific guidance by proposing effective and sustainable solutions to address the ecological challenges presented by *Limnoperna fortunei*. The insights gained from current research not only reveal novel antifouling methods, but also identify key areas for further investigation aimed at enhancing performance and environmental compatibility.

## 1. Introduction

Biofouling is a prevalent natural phenomenon that involves various organisms, including microorganisms (such as bacteria and fungi), algae, plants, and invertebrates, which attach and grow at the bottom of ships, underwater buildings and other underwater facilities. These attachments not only cause changes in the appearance of the facility, but also have a significant impact on its performance, operational efficiency, and service life [1,2]. The formation process of biofouling is common in high-salinity marine environments and relatively low-salinity freshwater ecosystems, but there are significant differences in the specific biological composition and interaction mechanisms. Especially in freshwater environments, the unique water quality, temperature, and lighting conditions provide ideal conditions for biological attachment and growth [3,4,5]. These factors collectively promote the rapid proliferation of freshwater biological communities, leading to particularly complex biofouling formation in freshwater environments. The interactions between microbial communities and large invertebrates, especially the freshwater bivalve *Limnoperna fortunei*, are particularly crucial. *Limnoperna fortunei*s, with its significant attachment and growth abilities, provides an ideal substrate for the attachment of other organisms by secreting mucus and forming biofilms, thereby promoting the formation of multi-layered biofouling structures. Therefore, when discussing the control of biological pollution caused by *Limnoperna fortunei*, it is essential to thoroughly consider the interaction and influence between microorganisms and *Limnoperna fortunei*.

As one of the most widespread freshwater invasive species, *Limnoperna fortunei* originates from the river systems of China and Southeast Asia [6,7]. It can be attached and grown in large numbers on the surfaces of underwater facilities, such as water conveyance structures, pipelines, and ship bottoms, which significantly poses a serious threat to the performance and safety of these facilities [8,9,10,11]. For example, *Limnoperna fortunei* can have a negative impact on aquatic ecosystems by facilitating the spread of invasive species and disrupting ecological balance. Additionally, it has the potential to affect water quality, for instance, by altering nutrient cycles involving nitrogen and phosphorus by certain microorganisms, thereby affecting the health and biodiversity of aquatic environments. Furthermore, the attachment of *Limnoperna fortunei* accelerates the corrosion process of underwater structures, reducing their durability and safety. In human activities, *Limnoperna fortunei* can increase vessel motion resistance, leading to higher fuel consumption and decreased operating efficiency [12,13]. The presence of *Limnoperna fortunei* in water treatment systems and hydroelectric stations has the potential to block pipelines and filters, lower system efficiency, promote metallic corrosion, and even shorten the lifespan of facilities [14,15,16].

Microbial fouling not only affects the appearance of materials, but also it can contribute to the corrosion process, as microbes produce acidic substances or engage in redox reactions through their metabolic activities. Moreover, microbial fouling can lead to the deterioration of water quality, posing threats to aquatic organisms and human health. Therefore, the design of antifouling materials often considers dual protection against both invertebrates and microbes. They prevent the attachment and growth of invertebrates and microbes through various mechanisms, such as physical and chemical interactions.

Antifouling coatings play an important role in reducing the issues caused by the attachment and fouling of *Limnoperna fortunei*. More research is needed to better understand the mechanics and long-term impacts of these coatings, as well as to explore the combination of different preventative approaches to improve efficacy [17,18,19]. However, the application of antifouling coatings or cleaning chemicals may lead to secondary pollution of aquatic environments. To contribute to sustainable development, these coatings must be environmentally safe and sustainable. Despite the availability of antifouling coatings on the market, they still face challenges related to environmental, durability, and cost-effectiveness concerns.

In this review, we focus on the control strategies for biofouling by *Limnoperna fortunei*, particularly the progress in research on antifouling coatings. Initially, we outline the unique biological characteristics, growth habits, attachment mechanisms, and widespread distribution of *Limnoperna fortunei* in aquatic environments. Subsequently, we systematically assess the advantages and disadvantages of conventional antifouling coatings, including physical, chemical, and biological methods, and discuss their potential environmental impacts and the requirements of environmental policies. Thereafter, we emphasize the research advancements in novel antifouling coatings, including eco-friendly coatings, biodegradable coatings, nano-antifouling coatings, and structured surface coatings, and provide a comprehensive evaluation of their antifouling effectiveness, environmental impacts, and cost control. Finally, we analyze the challenges and technical limitations currently faced in the development of antifouling coatings and provide insights into future research directions. Through this comprehensive review, we aim to provide theoretical support and practical guidance for the sustainable development of antifouling coating technologies.

## 2. Biological Characteristics of *Limnoperna fortunei*

### 2.1. Limnoperna fortunei

**Figure 1:**
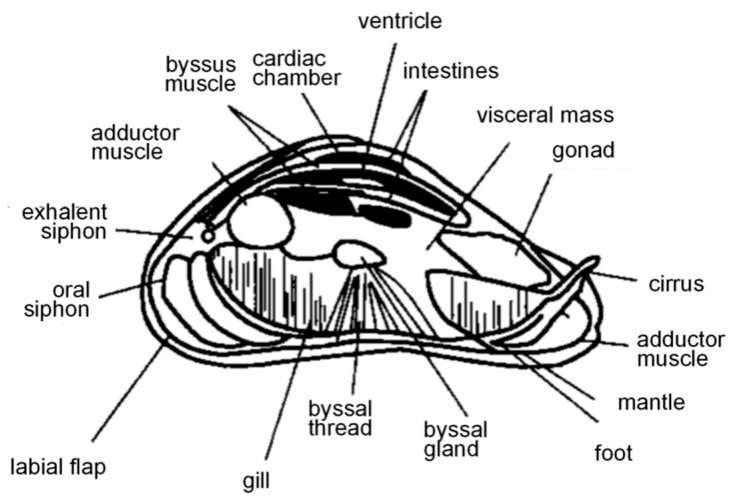
Internal structure of *Limnoperna fortunei* [20].

*Limnoperna fortunei*, commonly known as the golden mussel, belongs to the mollusk phylum, bivalvia class, and mytilidae family. This organism is a filter-feeding benthic organism and an important source of biofouling in freshwater environments. The body of mussel is nearly triangular, with shell lengths ranging from 20 to 25 mm and up to 60 mm, and a dorsal margin that forms a distinct arc with the posterior edge [20], as depicted in Figure 1. The color of the shell varies from brownish-tan and yellow-green to dark brown, with violet and light blue iridescence within the shell. *Limnoperna fortunei* can adapt to a wide range of complex ecological conditions, including water structures with low dissolved oxygen levels and high flow rates [21].

*Limnoperna fortunei* exhibits remarkable adhesive capability. Despite having a small foot, its byssal threads are highly developed, allowing for strong adhesion to various hard surfaces. Particularly, it produces adhesive proteins that rapidly solidify and produce strong attachment points. This characteristic allows *Limnoperna fortunei* to maintain a stable presence in environments with strong water currents, forming durable biofilms or aggregations on surfaces such as ship hulls, dams, water pipes, and water treatment and power generation facilities, resulting in significant biofouling within a short period. Additionally, the excessive growth of *Limnoperna fortunei* can disrupt natural balances in water bodies, and their disintegration after death can decrease water quality, thereby threatening the survival of other aquatic organisms.

### 2.2. Biological Characteristics

*Limnoperna fortunei* has a short lifespan, rapid growth, and strong reproductive capabilities. When it is grown, it becomes sessile, feeding mainly on organic particles, algae, and protozoans through filter-feeding with its gills [22]. The life cycle of *Limnoperna fortunei* consists of the following three stages:

(1) Larval Stage

During this stage, external fertilization occurs, with cell division completing the blastula and gastrula phases within a few hours, followed by the veliger larva, pediveliger, and juvenile stages. At this point, larvae float in the water, gradually developing shells and beginning to feed through gill respiration. They eventually settle and begin their sessile life, secreting byssal threads for attachment.

It is noteworthy that the larval stage is a critical period in the lifecycle of *Limnoperna fortunei*. During this period, the larvae possess a high dispersal ability, allowing them to spread to new aquatic environments via water flow. Their attachment behavior provides the foundation for subsequent biofouling. When larvae float in the water, they may encounter microorganisms, such as organic matter, algae, and other microbes. They may use these microbes as food sources to promote their growth and reproduction. The entire larval stage lasts about 30 days.

(2) Juvenile Stage

*Limnoperna fortunei* begins its juvenile phase following its attachment to hard surfaces via byssal threads. It reaches sexual maturity at a body length of approximately 8 mm, although growth rates vary by region. In South America, the shell growth rate is 8 mm/year, whereas in Asia, it reaches 15–20 mm/year [23].

This variation in growth rates not only reflects the impact of environmental factors on the growth potential of *Limnoperna fortunei*, but also suggests that regional growth differences should be considered when applying antifouling coatings in diverse areas.

(3) Mature Stage

*Limnoperna fortunei* reaches sexual maturity when its gonads are fully developed. The reproduction occurs when water temperatures are between 16 and 28 °C [24], with 1–2 reproductive cycles per year. This is primarily dependent on temperature [25]. In southern China, the breeding period can extend up to eight months and lasts from February to September. The lifespan of *Limnoperna fortunei* is limited by population density, with individuals living between 2 and 10 years. According to dissections, over a thousand juveniles were found within a single female [26]. This remarkable reproductive capacity further highlights its significance as a major source of biological fouling.

### 2.3. Growth and Attachment Mechanisms

The growth of *Limnoperna fortunei* is influenced by various environmental factors, including water temperature [27,28], light [29], dissolved oxygen [30], nutrient levels [31,32,33,34,35], flow velocity [36], and pH values [30]. They exhibit strong reproductive capabilities at temperatures between 16 and 28 °C, which allows them to grow rapidly. Lower temperatures can inhibit reproduction, while higher temperatures can disrupt metabolism and increase mortality rates. The ideal pH range for their aquatic environment is between 6 and 8 [37].

The attachment process for *Limnoperna fortunei* begins in the larval stage, marking a crucial phase in its life cycles that significantly contributes to its biofouling potential. The larvae of *Limnoperna fortunei* quickly identify suitable hard surfaces when they enter aquatic environments. This selection process is facilitated by their ability to detect and interact with microbial biofilms, which act as primary points for initial attachment. After recognizing the surface biofilms, the pedal gland releases adhesive substances for preliminary fixation. This process depends heavily on adhesive proteins, which quickly solidify under water and form strong chemical bonds with the substrate. This ensures a strong attachment to various hard surfaces, such as concrete.

**Figure 2:**
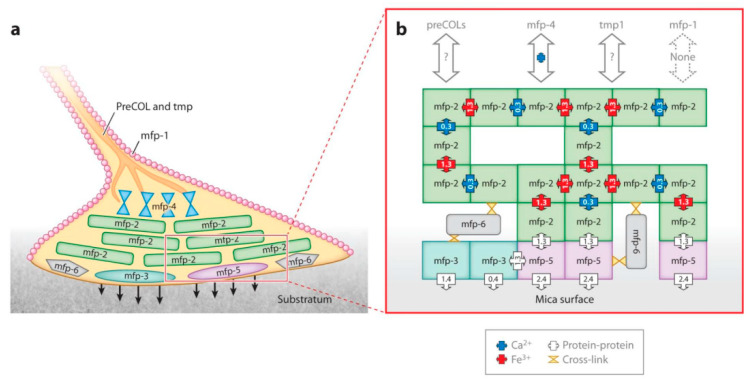
Schematic view of mytilus edulis foot proteins in a byssal plaque. (**a**) Mefp-3 variants and mefp-5 are thought to be the adhesives (arrows). (**b**) Schematic view of all known mefp interactions as determined by the surface forces apparatus [39].

Mytilus edulis foot proteins (Mefp) are the primary components of the mucus secreted by the byssal threads of *Limnoperna fortunei*. Figure 2 illustrates the schematic representation of Mefps in the byssal plaque and their interactions [38,39]. The principal components of byssal thread proteins are glycoprotein-type mucins, including at least eight protein types: Mefp-1, Mefp-2, Mefp-3, Mefp-4, Mefp-5, Mefp-6, PreCol-P, PreCol-D, and PreCoL-NG [40,41]. Table 1 lists detailed information about their molecular weights and functions.

As depicted in Table 1, the mussel’s byssal threads are composed of numerous proteins, each playing a specific role. The mussel adhesive protein is rich in 3,4-dihydroxyphenylalanine (DOPA), which is responsible for its strong adhesive properties. The foot glands of the mussel secrete when they contact a solid substrate. The hydroxyl groups of DOPA can form hydrogen or coordinate bonds with the substrate, thereby anchoring to the surface of the material. The primary contributor to adhesion is the Mefp-5 protein, with DOPA being the key functional unit within it that facilitates attachment behaviors [22,23]. The DOPA side chain of amino acids contains catechol (orthodihydroxybenzene) functional groups, which give it multiple chemical capabilities and distinctive properties [41]. The byssal adhesive proteins rich in DOPA possess adjacent hydroxyl groups (-OH) that are reactive and capable of forming hydrogen, covalent, and ionic bonds with various substrates (e.g., concrete), thus achieving effective adhesion [45]. Moreover, the process of attachment is influenced by physical factors, such as surface roughness and flow rates [51,52]. The surface properties and microstructure of the substrate have a significant influence on the attachment efficiency and subsequent growth of *Limnoperna fortunei*. Areas with slower flow rates typically exhibit higher levels of bivalve attachment, whereas the opposite is observed in areas with faster currents.

### 2.4. Habitat and Distribution of Limnoperna fortunei

**Figure 3:**
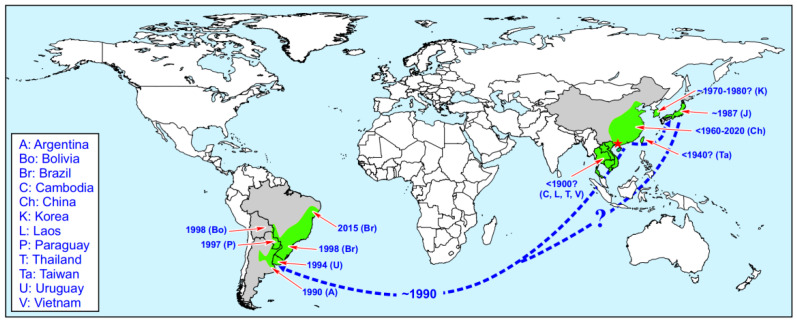
Worldwide invasive ranges of *Limnoperna fortunei*. Red star denotes native area (the Pearl River Basin, China). Gray area denotes countries invaded; light green: areas invaded (in some cases, as in Indochina and Korea, presence of the mussel is restricted to some basins). Blue arrows denote probable entry routes. Labels denote approximate years of introduction and country/region names [7].

*Limnoperna fortunei* is primarily found in temperate and subtropical freshwater lakes and rivers, exhibiting a remarkable tolerance to salinity variations. Several factors impact the distribution of this species, including climate, water quality, and food availability. In China, for example, *Limnoperna fortunei* is found in the middle and lower reaches of the Yangtze River and regions to the south, including provinces such as Hunan, Hubei, Jiangxi, Anhui, Jiangsu, Guangdong, Guangxi, Zhejiang, and Fujian. After its spread to South America in the 1990s, *Limnoperna fortunei* has reproduced at remarkable rates, especially within the Amazon River basin [53]. It is found in five South American countries, including Brazil, and poses a significant ecological invasion threat to local ecosystems. The global invasive ranges of *Limnoperna fortunei* are illustrated in Figure 3.

## 3. Antifouling Mechanisms of Coatings

The issue of biological fouling by *Limnoperna fortunei* represents a significant global challenge in aquatic environments, not only due to their considerable biomass causing direct damage to under water structures, but also due to the synergistic effects of microbial fouling. In order to address this challenge, researchers have explored three primary categories of control strategies: physical, chemical, and biological.

Chemical methods, such as using chlorine and sodium hypochlorite, have shown some effectiveness in controlling micro fouling [54]. However, their limitations are evident when dealing with large invertebrates like *Limnoperna fortunei*. Furthermore, certain chemical agents present in the antifouling coating may undergo degradation or leaching over time, thereby reducing their effectiveness. Moreover, the long-term application of these coatings may lead to the development of certain drug resistance in aquatic organisms. Additionally, owing to the sensitivity required for chemical selection, the precision required in dosing, and the potential for secondary water quality pollution, the implementation of chemical methods is strictly regulated.

Biological methods utilize the inherent predation behaviors of aquatic organisms, such as fish [55], to control the growth and reproduction of *Limnoperna fortunei*. This approach avoids the use of chemical agents and exhibits minimal impact on aquatic ecosystems. Furthermore, it can provide additional ecological and economic benefits. However, the antifouling effectiveness of these approaches is affected by virious factors, such as the selection of biological species and the type and quantity of natural enemies, as well as environmental factors, such as water temperature, water quality and food availability. This results in variable and sometimes unreliable control effects, which may not meet the demands of emergency prevention and control.

Physical methods include manual scraping, ultraviolet light exposure, ultrasonic treatment [56] and the application of antifouling coatings. The manual scraping method is a direct and effective approach to removing *Limnoperna fortunei* attachment. Although this technique is notably straightforward and cost-effective, it requires significant physical labor, making it labor-intensive and potentially less efficient in achieving complete removal. UV irradiation provides a chemical-free solution for controlling *Limnoperna fortunei*. By utilizing the power of ultraviolet light, this method can effectively eliminate the *Limnoperna fortunei* with minimal environmental impact. However, this method is time-consuming and less effective for removing *Limnoperna fortunei* that are deeply embedded or obscrued. Ultrasonic treatment provides an efficient and fast method without chemical pollution. It utilizes the cavitation effects of ultrasound to disrupt the attachment of *Limnoperna fortunei* to their substrates. Nonetheless, the implementation of this technology is hindered by the high cost of the equipment and the necessity for regular maintenance by trained professionals.

Recent research has focused on antifouling coatings due to their relatively simple application, minimal environmental disturbance and durable antifouling capabilities. These coatings are often designed with unique surface characteristics, such as low surface energy and superhydrophilic properties, or they incorporate functional additives like nanoparticles to effectively prevent the attachment and growth of *Limnoperna fortunei*. These antifouling coatings have a relatively minimal impact on aquatic ecosystems, and their antifouling effect can last for a long time. This reduces the requirement for frequent replacement of maintenance. Nonetheless, the preparation of these coatings with special structures requires advanced technology and cost, as well as precise control of construction. The effectiveness of these coatings primarily depends on the stability and persistence of the surface structure, as well as the adhesion mechanism and growth habits of *Limnoperna fortunei*. Furthermore, it is also impacted by diverse factors such as the coating thickness, uniformity, aging degree, quantity and growth process of *Limnoperna fortunei*.

It is well-known that the biofouling caused by *Limnoperna fortunei* is not an isolated issue. Its control is closely related to broader strategies for microbial fouling management. The attachment of microorganisms to the surface of materials provides an important foundation for the initial and subsequent attachment of *Limnoperna fortunei*. Therefore, the study of the control mechanisms of microbial fouling provides an important perspective for understanding the attachment mechanisms of *Limnoperna fortunei*.

### 3.1. Conventional Antifouling Coatings

Conventional antifouling coatings primarily rely on the release of toxic substances in order to prevent the attachment of organisms such as *Limnoperna fortunei*. These coatings often contain active ingredients that inhibit or kill adhesive organisms, such as heavy metals (copper, silver, zinc, etc.) [57,58,59,60] and organotin compounds, including tributyltin [61,62,63,64]. These toxic substances possess the capacity to disrupt cellular structures or disrupt physiological functions of the biofouling organisms, thereby achieving their antifouling effect. Furthermore, certain conventional coatings enhance the difficulty of biological attachment by altering the physical characteristics of the coating surface, such as the creation of ultra-slippery or micro-structured surfaces [65,66,67]. These designs prevent the formation of stable biofilms and the effective adhesion of coating surfaces.

However, the duration of effectiveness of antifouling agents containing heavy metals is constrained by several factors, including the dissolution rate of the heavy metals, coating thickness, water quality conditions (such as hardness, pH, and dissolved oxygen content), and the tolerance of *Limnoperna fortunei*. As the heavy metal ions are slowly consumed and diluted in the environment, their effectiveness gradually diminishes. Typically, these coatings can provide effective protection ranging from several months up to a few years.

#### 3.1.1. Environmental Impact and Limitations of Conventional Antifouling Coatings

Although conventional antifouling coatings are effective in preventing the attachment of organisms like *Limnoperna fortunei*, they also pose significant environmental hazards.

(1) Biotoxicity: Conventional coatings contain heavy metals and organotin substances that are highly toxic to aquatic life [68]. These toxic components gradually disperse into the aquatic environment, affecting the reproduction and growth of aquatic organisms and potentially leading to the long-term degradation of some species.
(2) Bioaccumulation: Toxic substances released into the environment can accumulate in the food chain, eventually affecting higher-level consumers, including humans.
(3) Ecological Disruption: The presence of harmful components in conventional coatings may disperse through water currents, potentially harming the entire aquatic ecosystem and threatening the survival and reproduction of other organisms.

Given the environmental risks associated with conventional antifouling coatings, many countries and regions have implemented stringent regulations in order to restrict or prohibit the use of certain toxic chemicals. These measures aim to reduce the ecological impact of these substances, ensuring a balance between effective biofouling prevention and environmental conservation.

#### 3.1.2. Regulatory and Policy Environment

The potential environmental hazards associated with conventional antifouling coatings have become a major concern for international organizations and governments. Given the significant impacts of biofouling organisms like *Limnoperna fortunei* on the utilization of water resources, the safety of hydraulic facilities, and ecological environments, research and the application of specific antifouling coatings are particularly critical.

The International Maritime Organization (IMO) primarily regulates marine antifouling coatings through Annex VI of the International Convention for the Prevention of Pollution from Ships [69], which prohibits the use of toxic substances in conventional antifouling paints. The development of eco-friendly antifouling coatings has been sparked by this regulation, which not only prevents the attachment of *Limnoperna fortunei* but also minimizes potential environmental dangers. The European Union has further refined its restrictions on the use of antifouling products containing specific chemicals in both marine and freshwater environments, promoting the research and application of non-toxic, effective, and eco-friendly antifouling coatings for *Limnoperna fortunei* [70]. The U.S. Environmental Protection Agency has issued strict guidelines regarding the chemical substances employed in ship coatings, which will facilitate the sustainable growth of the *Limnoperna fortunei* [71]. The Chinese government has also responded to international environmental trends in recent years by issuing specific regulations aimed at preventing biofouling from organisms like *Limnoperna fortunei*, imposing strict limits on harmful components in antifouling coatings [72].

These measures not only protect the health of freshwater ecosystems, but they also progressively steer research and application of antifouling coatings for *Limnoperna fortunei* towards more environmentally friendly, effective, and sustainable directions. This provides a robust safeguard for addressing biofouling concerns in freshwater ecosystems.

### 3.2. Antifouling Mechanisms of Novel Coatings

#### 3.2.1. Eco-Friendly Coatings

Owing to the limitations of conventional antifouling coatings in terms of environmental protection and performance, eco-friendly coatings avoid the use of heavy metals and organotin compounds, opting instead for safer chemicals or ingredients derived from natural sources, aiming to minimize adverse effects on aquatic ecosystems during the antifouling process [73]. The antifouling mechanisms of these coatings involve several factors, including:

(1) Natural Antifouling Compounds

These coatings use antifouling active ingredients derived from marine organisms and plants [74,75,76,77,78]. Antifoulants, such as terpenoid sugars [79,80], halogenated furanones [81,82], capsaicin [83,84], and monoterpene phenol [85], can be effective in inhibiting the attachment and growth of freshwater bivalves, while being harmless or low-toxic to humans and the environment. However, their low content in organisms, complex extraction processes, and poor stability can lead to a loss of antifouling functionality. Table 2 shows the mechanism and the sources of the various types.

(2) Low Surface Energy Materials

The surface energy and contact angle of materials are two important parameters to consider in the development of antifouling coatings. Materials with low surface energy [95,96], such as silicone and fluorocarbon resins [97], can effectively reduce the surface free energy of coatings, thereby weakening the wetting and adhesion of water molecules and fouling organisms (including *Limnoperna fortunei* and their attached microbial communities) on the surface. This change in physical properties is the initial stage of biofouling. Under the action of natural forces, such as water flow, these attachments are more prone to detachment, which maintains the cleanliness and long-term antifouling performance of the coating. Moreover, materials with low surface energy also exhibit the ability to inhibit the growth and reproduction of microorganisms, which further enhances the comprehensive antifouling ability of the coating. This makes it possible to maintain a stable antifouling effect in freshwater environments for a long time.

The contact angle is an important parameter for measuring the degree of wetting of a liquid on a solid surface. In the field of antifouling, materials with low surface energy typically exhibit significant hydrophobic characteristics and larger contact angles, which makes it difficult to be wetted by fouling organisms. Low surface energy coatings can effectively inhibit the formation of microbial films, as the adhesion and reproduction of microorganisms also depend on the wettability and accessibility of the surface.

**Figure 4:**
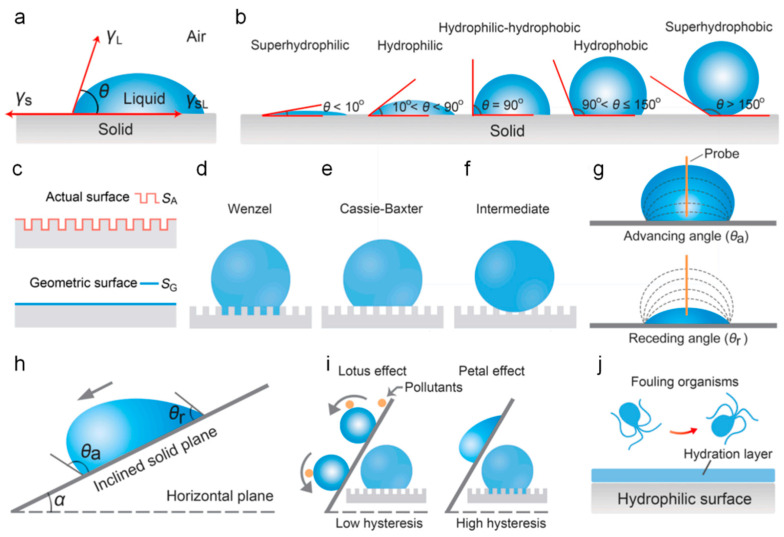
(**a**) Schematic illustration of the definition of the static contact angle. (**b**) The different states of a droplet in contact with a solid surface. (**c**) Actual surface and geometric surface areas. (**d**) Wenzel state. (**e**) Cassie-Baxter state. (**f**) Intermediate state. (**g**) Schematic illustration of the advancing angle (*θ*_a_) and receding angle (*θ*_r_). (**h**) Dynamic contact angle on a tilted surface. (**i**) Lotus effect and petal effect. (**j**) Antifouling mechanism of a hydrophilic surface [98].

The relationship between the contact angle and the wettability of a droplet on the surface of the material is illustrated in Figure 4a,b. Therefore, it is essential to understand the contact angle in order to study the antifouling mechanisms of materials. In Figure 4a, *θ* represents the contact angle, also referred to as the static contact angle, which is the angle between the liquid-gas and liquid-solid interfacial tensions. Based on the *θ* value, surface properties can be categorized as follows: superhydrophilic (*θ* < 10°), hydrophilic (10° < *θ* < 90°), hydrophilic-hydrophobic (*θ* = 90°), hydrophobic (90°< *θ* < 150°), and superhydrophobic (*θ* > 150°), as depicted in Figure 4b. For rough surfaces, the actual area (*S_A_*) and the projected area (*S_G_*) are shown in Figure 4c, where the contact angle *θ*_w_ relates to *θ* as follows: $cos\;\theta_{w}=rcos\;\theta$, $r=S_{A}/S_{G}$. When the droplet completely fills the gaps in the rough surface, it forms the Wenzel state, as illustrated in Figure 4d. When the gaps are filled with air, the droplet rests on both the solid and the air, known as the Cassie-Baxter state, as shown in Figure 4e. There exists an intermediate transitional state where the droplet partially fills the surface gaps, as depicted in Figure 4f. In nature, material surfaces are typically not uniformly smooth and level, and droplets on them are not static. It is necessary to investigate the dynamic contact angle using the detection techniques illustrated in Figure 4g. When a droplet is placed on the surface of the material, liquid is continuously added to increase its volume. When the volume reaches a critical value, the droplet begins to move forward. At present, the contact angle is referred to as the advancing angle *θ*_a_. Similarly, if liquid is withdrawn from the droplet and its volume decreases to a certain level, the droplet begins to move backward, at which point the contact angle is called the receding angle *θ*_r_.

The superhydrophobic surface of lotus not only hinders water droplets from wetting the surface, but also facilitates the rolling of droplets and the self-cleaning of contaminants through its distinctive dynamic contact angle characteristics [98], including advancing and receding angles, as shown in Figure 4h. When the sliding angle is small, water droplets can easily carry contaminants away from the surface during rolling, achieving efficient self-cleaning. The petal effect, which involves robust adhesion, allows droplets to persist on the petal surface. However, due to its high adhesiveness, it fails to effectively eliminate contaminants, as depicted in Figure 4i. This indicates that low contact angle hysteresis plays a crucial role in facilitating the removal of biological fouling on superhydrophobic surfaces.

However, when materials with small contact angles exhibit hydrophilicity, the antifouling mechanism is distinct from the lotus effect. An antifouling barrier is established by the formation of a hydration layer through hydrogen bonding or electrostatically induced hydration. As shown in Figure 4h, this hydration layer provides a physical barrier against the adhesion of contaminants, effectively preventing their direct contact and adhesion. This mechanism of antifouling on hydrophilic surfaces provides new insights for the development of novel antifouling coatings.

It is noteworthy that the biofouling of *Limnoperna fortunei* is frequently associated with a synergistic effect of microbial fouling. Microorganisms play an important role at both the initial and later stages of attachment to *Limnoperna fortunei*. They improve the stability of *Limnoperna fortunei* adhesion and may form biofilms on their surfaces, which serve as refuges for other fouling organisms. Therefore, when designing antifouling coatings, it is essential to consider a dual inhibition strategy targeting both *Limnoperna fortunei* and its associated microbes. This comprehensive approach is essential for achieving long-lasting antifouling effectiveness and maintaining high performance in diverse aquatic environments.

(3) Enzyme Inhibitors

**Figure 5:**
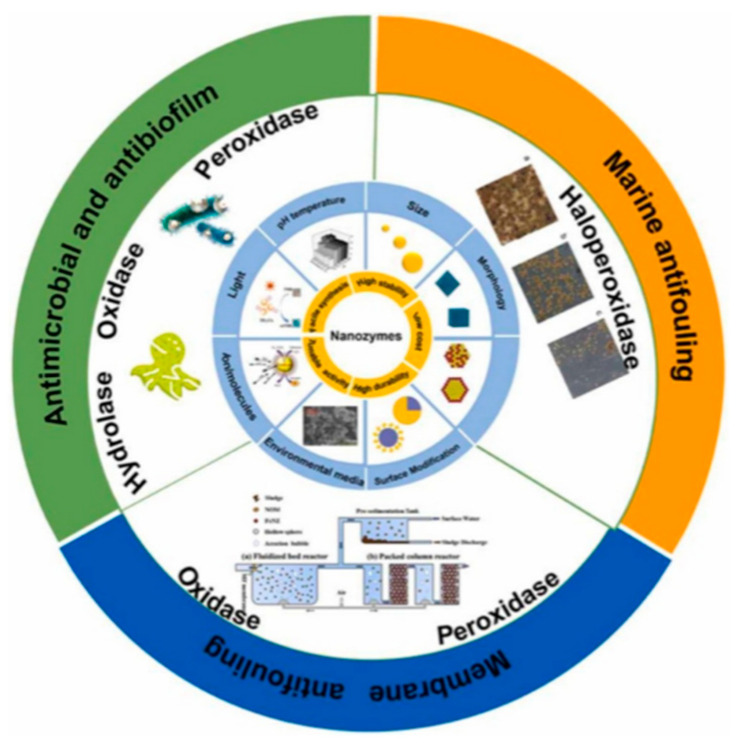
Classification and application of enzymes [100].

Nanoenzymes have received widespread attention for their antimicrobial and antifouling properties due to their inherent high stability and good adaptability to environments. By modifying the morphology, size, and compositional catalytic activity of nanoenzymes, their biological activity can be effectively controlled. Coatings with enzyme inhibitors can block or disrupt crucial stages in biofilm formation, thereby effectively preventing biological attachment. Wang et al. have developed a mono-molybdenum photothermal nanoenzyme with HPO activity that selectively catalyzes the oxidation of Br^-^ to form bactericidal HOBr, thereby significantly inhibiting biofilm formation [99]. The classification and application of enzymes are depicted in Figure 5.

The duration of coatings that employ enzyme inhibitors is determined by their stability and release rate of the inhibitors, as well as by the sensitivity of the *Limnoperna fortunei* to these inhibitors. If the enzyme inhibitors remain stable within the coating and continue to be released into the environment, then their effectiveness may be prolonged. However, the stability and release characteristics of the enzyme inhibitors can be affected by light exposure, temperature, and water flow rate. Generally, antifouling coatings containing enzyme inhibitors can maintain their protective effects for several months to about one year.

#### 3.2.2. Biodegradable Coatings

Biodegradable coatings utilize polymers that can be decomposed by environmental microorganisms as a substrate. This reduces environmental residues after the effective period of the coating. Their mechanism mainly reflects in two areas:

(1) Biodegradability

**Figure 6:**
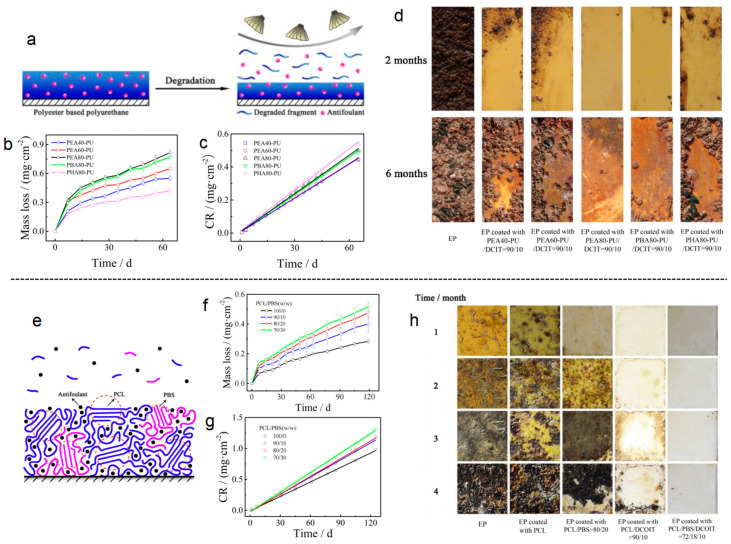
(**a**) The mechanism of degradable polyurethane. (**b**) Time dependence of mass loss of the polyurethane in ASW at 25 °C. (**c**) Time dependence of the cumulative release (CR) of DCOIT from the polyurethane. (**d**) Coating surface with different degradable polyurethane after immersion in the seawater [105]. (**e**) The mechanism of PCL/PBS blend. (**f**) Time dependence of mass loss of PCL/PBS blend in ASW at 25 °C. (**g**) Time dependence of the cumulative release (CR) of DCOIT from PCL/PBS blend. (**h**) Images of panels after immersion in the sea with different samples [106].

Polymers used in the coatings, such as polylactic acid (PLA) [101], polyurethane (PU) [102,103], and polycaprolactone (PCL) [104], exhibit a gradual degradation under the influence of microorganisms through photodegradation, oxidation, and hydrolysis. These coatings can control the release rate of eco-friendly antifoulants and exhibit excellent antifouling performance and renewability. The mechanisms of the degradable polymer and the coating surface after immersion in the sea are illustrated in Figure 6.

The duration of the impact of these coatings is closely associated with the degradation rate of the coating and the life cycle of the *Limnoperna fortunei*. Although these coatings are generally safe, the degradation process is influenced by various environmental factors, such as ultraviolet radiation and water erosion. These factors may result in varying protective effects of the coatings depending on the environmental conditions. Generally, well-designed biodegradable coatings are capable of providing effective antifouling protection over an extended period, ranging from several months to several years.

(2) Controlled Release Technology

The coating may contain natural extracts or synthetic compounds with antifouling effects. These components are gradually released during the degradation process of the coating [107,108,109], thereby ensuring its effectiveness in preventing the formation of antifouling for a long time.

**Figure 7:**
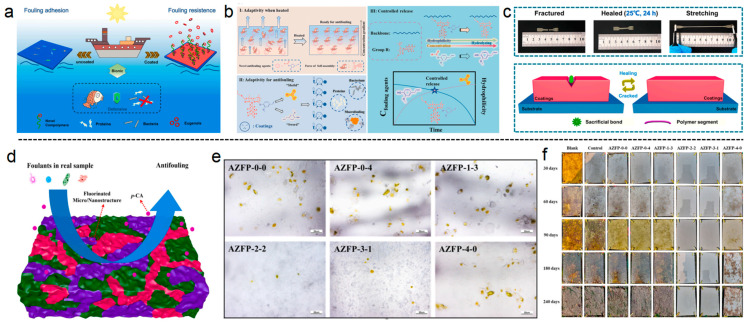
(**a**) Carp-inspired antifouling coating for ships with robust controlled release of eugenol. (**b**) Mechanism of controlled release of eugenol. (**c**) The cut-off and healed PDMS-Pun-5 sample and schematic diagram of self-healing mechanism [108]. (**d**) Antifouling mechanism of the AZFP. The surface is the combination of fluorinated regions (hydrophobic, green), carboxylic acid groups (hydrophilic, purple), and the p-CA (hydrophilic, pink). (**e**) Optical microscope images of AZFP after immersion in chlorella suspension for 8 d. (**f**) Images of the samples after immersion in the Yellow Sea for 240 d [110].

Liu et al. prepared a self-regulating marine antifouling coating with the ability to control the release of eugenol and efficient self-healing performance [108], as shown in Figure 7a. The controlled release mechanism is related to the special adaptability of the coating, and the release of eugenol is related to the alkaline marine environment, the hydrophilicity of the coating, pH value, etc., as shown in Figure 7b. The dumbbell shaped coating exhibits self-healing ability at room temperature within 24 h, and the self-healing mechanism is depicted in Figure 7c. Yu et al. have prepared an acrylate zinc fluorinated polymer, which can be hydrolyzed in seawater to slowly release the green antibacterial p-coumaric acid and form hydrophobic properties on the micro/nanostructure surface [110]. Marine field tests have demonstrated that these coatings exhibited an excellent static antibiofouling performance for over 240 days, as depicted in Figure 7d–f. The collaborative antifouling mechanism provides a new system strategy for the development of novel green, environmentally friendly, and efficient antifouling coatings.

Seawater, including various salts, microorganisms, and dissolved oxygen, plays a significant role in the degradation and corrosion of antifouling coatings. Although freshwater has a lower salt content and may contain fewer types and quantities of microorganisms compared to seawater, it still possesses a specific decomposition ability for controlled release coatings. This exhibits a certain similarity in the mechanism of antifouling with differences in degree and rate. Therefore, when designing a released antifouling coating, it is essential to consider their adaptability in different aquatic environments.

This coating technology can significantly improve the utilization efficiency of antifouling coatings and extend their duration of impact, maintaining their effectiveness over an extended period (such as several years). However, the design and manufacturing complexity of controlled-release systems is substantial, and it is necessary to comprehensively consider the environmental conditions and the biological characteristics of the *Limnoperna fortunei* in order to ensure their effectiveness and stability.

#### 3.2.3. Nano-Antifouling Coatings

Nanotechnology is the process of incorporating nanomaterials into antifouling coatings in order to enhance their overall performance and efficiency [59,111]. These nanotechnology techniques include:

(1) Nano-filler Effect

The incorporation of nanomaterials such as nano-silver [112,113,114], nano-copper [115,116,117], and graphene [118,119,120] into the coatings can significantly enhance their antibacterial, mechanical strength, and corrosion resistance, thereby enhancing the overall antifouling performance of the coatings.

**Figure 8:**
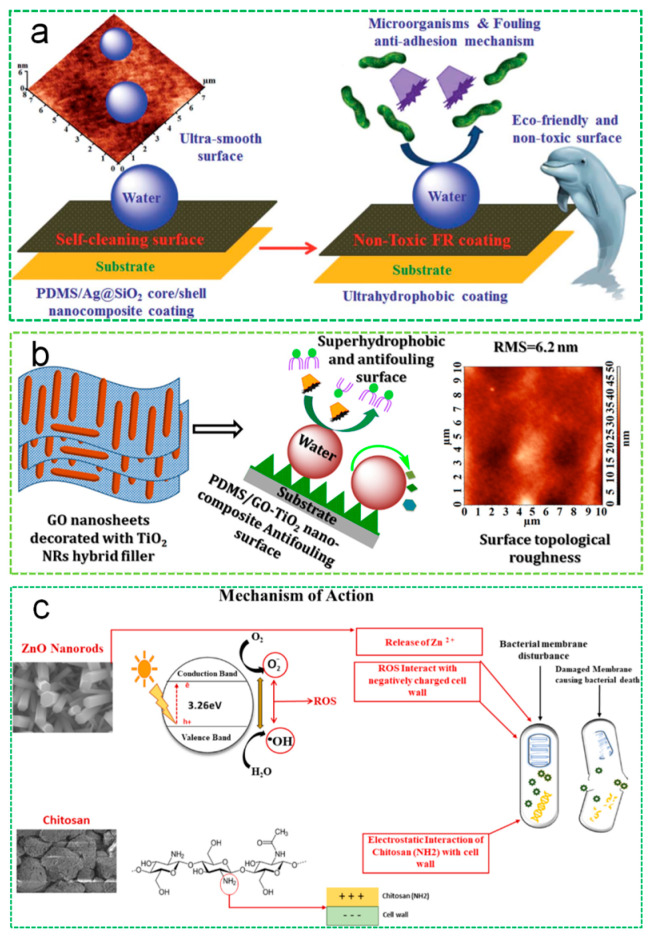
(**a**) The surface of PDMS/Ag@SiO_2_ core/shell nanocomposite coating and their failure adhesion mechanism [112]. (**b**) Mechanism of silicone/GO-TiO_2_ nanocomposite and surface topological of coating [121]. (**c**) Mechanisms of action of CHT/ZnO nanorod composite coatings [125].

Selim et al. studied the effect of Ag@SiO_2_ core-shell nanofiller on the self-cleaning and antifouling properties of silicone-based coatings [118], as depicted in Figure 8a. The addition of nanofiller enhanced the durability and tensile performance of the coating, which was primarily attributed to the nano-sized Ag particles inhibiting bacterial DNA replication and protein deactivation. Moreover, Selim et al. investigated a GO/TiO_2_ modified PDMS coating. With a concentration of 1 wt % GO/TiO_2_ nanofiller, the coating exhibited uniform topographic roughness, maximum superhydrophobicity, minimal surface free energy, and minimal dirt adhesion, which improved its thermal performance and mechanical properties. This composite coating exhibited optimal anti-fouling performance, as shown in Figure 8b [121].

(2) Photocatalytic Effects

Nanomaterials with photocatalytic properties, such as nano zinc oxide [122,123,124,125] and nano titanium dioxide [126,127], generate highly oxidative radicals under light exposure, which kill or inhibit organic substances and microbes on the coating surface. This ensures the cleanliness of the coating. Al-Belushi and colleagues incorporated ZnO nano-rods into a chitosan coating, which enhanced the antifouling activity of the composite coating. This is likely attributed to the generation of reactive oxygen species and the release of Zn^2+^ from ZnO nanorods, as shown in Figure 8c [125].

In summary, the strategic application of nanomaterials in antifouling coatings not only enhances the physical and chemical properties of the coatings, but also introduces functional capabilities that prevent biofouling through both mechanical resilience and active biochemical effects. This approach significantly improves the protection of coatings in freshwater environments.

#### 3.2.4. Structured Surface Coatings

Structured surface technology involves the creation of micro- and nano-scale surface structures that resemble natural antifouling strategies, such as shark skin and lotus leaf surface properties, in order to reduce the adhesion of organisms [128,129,130]. The antifouling mechanisms for these structured surfaces include:

(1) Microtextural Effect

**Figure 9:**
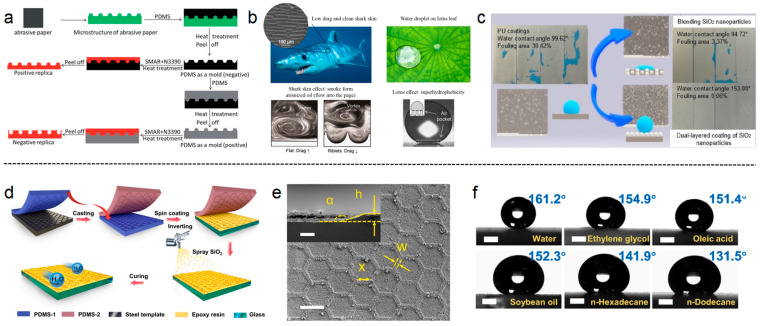
(**a**) The creation of microtextured coating from the abrasive paper [128]. (**b**) The scheme of surface structured coating: (**a**) shark skin and lotus effect mechanism [131]. (**c**) The contact angle and fouling area of PU and PU/SiO_2_ coatings [134]. (**d**) Schematic illustration of the fabrication of superhydrophobic coating with honeycomb structure via template and spraying methods. (**e**) FESEM image of superhydrophobic coating with honeycomb structure. (**f**) The contact angle of different droplets on hc-100-20 [133].

By creating specific tiny protrusions or grooves structures on the coating surface, these carefully designed textures reduce the actual contact area between organisms and the coating surface, thereby diminishing the attachment capability of freshwater bivalves. Chen et al. employed sandpaper to prepare a series of modified silicone coatings with varying textures, shapes, and surface roughness, discussing the impact of surface morphology and wettability on the antifouling performance of textured coatings [128]. The characteristic dimensions of surface morphology can effectively inhibit algal settlement through the “bridging” adhesion behavior, thus reducing the contact area and adhesion strength between the coating surface and algae, as shown in Figure 9a. Combining the contributions of surface texture and low surface energy performance, the construction of non-toxic and environmentally friendly antifouling coatings provides ideas for the biological control of *Limnoperna fortunei*.

(2) Superhydrophobic Properties

Through employing a structured surface design, coatings can achieve superhydrophobic characteristics [131,132,133,134]. The unique scale structure and anisotropic flow properties of shark skin effect can be used to create anti-fouling coatings that reduce the adhesion and drag of aquatic contaminants on the coating surface. Due to the superhydrophilic and self-cleaning properties of the lotus effect, coatings with similar micro-nano surface structures have been developed. These coatings prevent the attachment of freshwater biofoulers and facilitate their removal by water currents, as depicted in Figure 9b. Kanthasanmy et al. have enhanced the surface roughness of commercial polyurethane coatings by using silica to create a superhydrophobic surface [134]. The result indicates that changes in surface chemical properties are key factors affecting the antifouling performance of polyurethane coatings, as shown in Figure 9c. The design of the surface structure with antifouling facilitates the prevention of *Limnoperna fortunei* attachment to the antifouling coated surface and allows a self-cleaning effect under the action of water flow.

Li et al. prepared a honeycomb-structured superhydrophobic coating by using a templating and spraying technique [133]. They first patterned an epoxy resin coating to form micrometer-scale honeycomb-like ridge structures and then sprayed hydrophobic silica nanoparticles onto the honeycomb-like ridge structures before the epoxy resin fully cured. This resulted in a coating with high transparency and excellent wear resistance. The coating exhibits exceptional repellency against various liquids, including ethylene glycol and soybean oil. This provides effective anti-fouling and self-cleaning capabilities, as shown in Figure 9d–f. This wear-resistant and superhydrophobic coating helps prevent and control the fouling and adhesion of *Limnoperna fortunei*, and increases the service life of the antifouling coating.

In summary, utilizing the unique surface structures discovered in nature that possess antifouling mechanisms, researchers have successfully designed an efficient and environmentally friendly antifouling coating by constructing similar surface structures. The exceptional stability of these coatings in aquatic environments significantly extends their effective lifespan and reduces the frequency of maintenance and replacement, which in turn reduces the overall cost of use. Moreover, due to their environmentally friendly properties, these antifouling coatings minimize the potential threat to aquatic ecosystems. However, achieving such precise reproduction of microstructure is not without challenges. Highly precise manufacturing processes and tools are required. This adds complexity to the research and development phase and also increases the costs associated with production. Furthermore, as this innovative technology progresses from the laboratory to large-scale production, the challenges of standardizing production procedures and ensuring the continuous stability of product quality present significant challenges.

The comparison and summary of different coating strategies can be effectively presented in Table 3. Although conventional antifouling coatings are cost-effective in the short-term, their environmental impact and frequent need for reapplication decrease their long-term viability. In contrast, novel approaches, such as eco-friendly, biodegradable, and nano-antifouling coatings, offer improved sustainability and effectiveness, but at higher initial costs. Structured surface coatings exhibit minimal environmental impact and an innovative approach to fouling prevention, though their physical durability needs to be enhanced to ensure long-term effectiveness. By arranging the information in this table, we can quickly compare the different coating strategies and make informed decisions based on their specific needs and priorities.

## 4. Conclusions

*Limnoperna fortunei*, which is known for its strong adhesive capabilities and resilient vitality, has become a major challenge in the maintenance of hydraulic structures and water conveyance systems due to its propensity for causing biological fouling. This review summarizes a comprehensive overview of current research and emerging innovations in antifouling coatings, specifically designed to mitigate the effects of *Limnoperna fortunei*. Our analysis includes an exploration of the growth and adhesion mechanisms of *Limnoperna fortunei* and a detailed examination of the underlying principles of various antifouling strategies.

Recent advancements in this field indicate that antifouling technology has achieved notable success in addressing the complications associated with biofouling. For example, in an aquaculture reservoir in SP-Brazil, antifouling siginficantly inhibits the growth of *Limnoperna fortunei* [75]. Herein, we present state-of-the-art approaches that integrate both chemical and physical deterrents to prevent the attachment of *Limnoperna fortunei*. These include the development of eco-friendly, biodegradable, and nano-structured coatings, each of which has unique advantages and poses distinct challenges in terms of efficacy, environmental impact, and cost-effectiveness.

In conclusion, continued research and refinement of antifouling coatings are imperative. The continuous evolution of antifouling technology aims not only to improve the performance and sustainability of these solutions but also to foster a deeper understanding of the ecological interactions influenced by these interventions. To ensure the long-term protection of aquatic infrastructures against the pervasive threat of *Limnoperna fortunei*, future research should focus on improving the durability, efficacy, and eco-compatibility of antifouling coatings.

### 4.1. Challenges

The byssal threads of *Limnoperna fortunei* possess strong adhesive capabilities, enabling them to form durable attachments on various material surfaces. Consequently, antifouling coatings designed to combat these mussels must exhibit exceptional anti-adhesion properties. Given the ability of *Limnoperna fortunei* to thrive in diverse aquatic and environmental conditions, these coatings must also demonstrate exceptional environmental adaptability. This includes maintaining stable antifouling effects across different water qualities and temperature ranges, as well as ensuring the principles of environmental sustainability in both manufacturing processes and final products.

It is a critical challenge to ensure the long-term durability of these coatings in natural aquatic environments, which are often subject to severe conditions. Factors such as water erosion in turbulent or high-flow regions have the potential to gradually diminish the integrity of coatings. Furthermore, UV radiation can deteriorate the chemical structure of the coatings, thereby reducing their effectiveness over time. To address these issues, ongoing research has focused on developing innovative materials and formulations that enhance the durability and effectiveness of antifouling coatings. More importantly, the adhesion strength between coatings and protective materials is one of the key factors in evaluating the long-term stability and durability of the coatings. Inadequate adhesion strength can lead to the detachment or peeling of the coating after extended exposure to aquatic environments, thereby diminishing its ability to resist biofouling. Therefore, when developing coatings, it is essential to carefully consider their compatibility and adhesive performance with specific protective structures.

Concurrently, there exists a perceived contradiction between the durability required for long-lasting protection and the desirable property of biodegradability, which aims to minimize environmental impact after application. Typically, biodegradable coatings are designed for short-term use under specific conditions, which emphasizes the necessity for a strategic balance between efficacy and environmental responsibility. It remains a formidable challenge to develop coatings that not only effectively prevent biofouling but also minimize ecological footprints.

### 4.2. Current Technological Limitations

The current antifouling coatings exhibit limited effectiveness in preventing the attachment of *Limnoperna fortunei*, particularly in complex freshwater environments. The diversity of biotic and abiotic factors inherent to these ecosystems can adversely affect the performance of these coatings. Furthermore, numerous conventional antifouling products contain harmful chemicals, such as heavy metals and organic pollutants. These substances pose significant risks because they have the potential to bioaccumulate in aquatic organisms, ultimately leading to detrimental effects on the entire aquatic ecosystem.

Additionally, while high-performance, eco-friendly coatings are available, they are often associated with higher costs. The economic factor can be a significant barrier to their widespread adoption and application in aquatic settings where *Limnoperna fortunei* is prevalent. The challenge lies in developing cost-effective and environmentally conscious antifouling solutions that do not compromise on performance or environmental integrity.

### 4.3. Balancing Sustainable Development and Ecological Safety

The development of the next generation of antifouling coatings needs to achieve a balance between effective biofouling mitigation and environmental protection. In order to minimize adverse impacts on aquatic ecosystems, it is imperative that researchers focus on developing eco-friendly, low-toxicity, or non-toxic antifouling coatings. Moreover, the manufacturing processes of these coatings should adhere to the principles of sustainable development by utilizing eco-friendly components and procedures.

The development of these innovations requires conducting thorough ecological impact assessments. These assessments are crucial in ensuring that the novel coatings, when applied in real-world applications, do not result in unintended detrimental effects on aquatic ecosystems. By ensuring the ecological compatibility and sustainability of antifouling technologies, we will not only enhance environmental protection, but also facilitate the long-term viability of these solutions in addressing biofouling challenges.

### 4.4. Future Research Directions

(1) Development of eco-friendly coating: Future studies should focus on innovating eco-friendly antifouling coatings derived from natural products, in order to minimize environmental and ecological impacts [109,135,136]. At the same time, we are investigating the long-term stability and durability of these materials to ensure that antifouling coatings can maintain their performance for a long time in practical applications. Furthermore, when developing new environmentally friendly coatings, a comprehensive ecological safety assessment is necessary. This includes assessing the environmental impact of these coatings throughout their entire lifecycle, from preparation to disposal, as well as their potential hazards to aquatic organisms and ecosystems. Future research should focus on longitudinal studies to observe the long-term effects of biodegradable coatings on mussel populations in different ecological regions. These studies will help validate theoretical predictions with empirical data, ensuring the ecological safety and effectiveness of these coatings.
(2) Advancement through nanotechnology: The utilization of nanotechnology has the potential to significantly enhance the antifouling performance of coatings. Research should be directed towards developing intelligent coatings with self-repairing properties [108,137,138,139] and multifunctional coatings [140,141]. The polydimethylsiloxane with low surface energy was combined with polyurethane, which forms the novel type of waterborne polyurethane coating with good antifouling and self-healing abilities through incorporating polydimethylsiloxane and disulfide bonds [139]. These advanced coatings should adapt their chemical properties in response to environmental conditions, thereby improving durability and antifouling efficacy. For instance, by designing coatings with a multi-layered structure, the synergistic effect of multiple functions can be achieved, enhancing the overall performance of the coating. Future research should aim to elucidate the response mechanisms of these intelligent systems, enhance their performance characteristics, and explore their potential applications in complex aquatic environments.
(3) Improving formulation and process: The focus should extend to improving the anti-adhesion properties, durability, and environmental performance of antifouling coatings through formulation and process innovations. Future research should concentrate on examining the interaction mechanisms between coatings and diverse substrates, developing universal coatings that are suitable for multiple substrates, and providing customized coating solutions for specific substrates.
(4) Interdisciplinary integration: There is a compelling need to integrate knowledge and technologies from materials science, environmental science, biology, and chemistry into one integrated approach. This interdisciplinary approach will accelerate the development of sophisticated antifouling strategies tailored to manage biofouling by *Limnoperna fortunei* and potentially other invasive species.

In summary, it is a significant challenge to develop an effective, economical, and eco-friendly antifouling coating for *Limnoperna fortunei* that is suitable for complex freshwater ecosystems. Although recent research on coatings designed to mitigate biofouling by *Limnoperna fortunei* has yielded notable advances, the reliance on a singular antifouling mechanism often fails to meet comprehensive practical requirements. The future of antifouling technologies depends on integrating diverse strategies to harness their collective advantages and counterbalance their individual deficiencies. This integrated approach ultimately leads to the development of multifunctional coatings. The key to progress is the synthesis of insights from materials science, environmental science, biotechnology, and chemistry. Such interdisciplinary collaboration is essential for the development of innovative and intelligent antifouling solutions that are both more effective and environmentally responsible. Advancements in these areas are expected to significantly improve the sustainable management of critical infrastructure, including hydroelectric facilities and water distribution systems, ensuring their safe and efficient operation in the face of biological challenges posed by *Limnoperna fortunei*.

## Figures and Tables

**Table 1:** Protein types, molecular weight (kDa), and functions of *Limnoperna fortunei*.

Protein	Molecular Weight /kDa	Functions	References
Mefp-1	~110	Forms a protective coating over the byssus threads, shielding them from aqueous dissolution and microbial degradation. Exhibits limited adhesion capabilities.	[42]
Mefp-2	~40/45	A primary structural protein of the attachment base, potentially involved in cross-linking with byssal pad adhesion proteins. Contains 5 mol% of DOTA.	[43]
Mefp-3	~5–7	Localized at the interface between the attachment base and the substrate, acting as the principal adhesive protein in bond formation.	[44]
Mefp-4	~70–80	An adhesion protein in the byssal pad, responsible for linking collagen-like proteins (such as preCoD) within the byssus threads to the adhesion proteins of the byssal pad.	[45]
Mefp-5	~9.5	Predominantly found at the junction between the attachment base and external materials, considered the main adhesive protein facilitating the bond between mussel byssal pads and solid external surfaces.	[46]
Mefp-6	~11.6	An adhesion protein present in the byssal pad.	[47]
PreCol-D	~80	Distal Collagen Prepolymers: These confer significant toughness and superior extensibility to the terminal regions of byssal fibers, enhancing their mechanical properties under stress.	[48]
PreCol-P	~95	Proximal Collagen Prepolymers: These polymers imbue the byssal fibers with both resilience and elasticity, crucial for maintaining fiber integrity during dynamic environmental interactions.	[49]
PreCol-NG	~76	Non-Gradient Collagen Prepolymers: Serve as connectors between PreCol-D and PreCol-P, forming the core structural framework of the byssal fibers.	[50]

**Table 2:** Summary of some natural antifoulants.

Types	Mechanism	Sources	References
Terpenes	Inhibition of mycelial growth or destruction of cell membrane structure	Secondary metabolites from sponge dendrilla antarctica	[79,80]
Halogenated furanone	Inhibiting the attachment of fouling organisms	Red alga	[81,82]
Capsaicin	Inhibit adhesion, aggregation and accumulation of chlorella vulgaris	Solanaceae plants	[86,87,88]
Carvacrol	Destruction of cell wall and membrane integrity	Phenolic essential oils from plants in the family Lamiaceae	[85]
Tannic acid	Deactivate enzymes and proteins	The rhizomes, bark, and seeds of various plants	[89,90,91]
Triphenyl compounds	Inhibit the growth of diatoms	Brown algae	[92]
Coumarin	Fluorescent antifouling and contact bacteriostasis mechanisms	Leguminous plants	[93]
Paeonol	Bacteriostatic activity	Ranunculaceae plants and Asclepiadaceae plants	[94]

**Table 3:** Comparison of antifouling mechanisms for various coatings.

Type	Antifouling Effectiveness	Environmental Impact	Cost Control
Conventional Antifouling Coatings	Significantly reduce biofouling in the short term but require frequent recoating.	Utilize toxic antifoulants, leading to considerable environmental pollution.	Initial investment is relatively low, but long-term maintenance costs are high.
Eco-Friendly Coatings	Provide long-lasting fouling resistance and reduce environmental pollution.	Use natural or low-toxicity components, environmentally friendly.	Research and development costs are high, but overall cost-effectiveness is favorable.
Biodegradable Coatings	Coatings naturally degrade, minimizing long-term pollution; controlled release technology provides sustained antifouling effects.	Good biodegradability, low environmental impact over their lifecycle.	High research and development costs, but reduce subsequent coating treatment and removal expenses.
Nano-Antifouling Coatings	Highly effective fouling resistance due to significant nanoscale effects.	Safety of nanomaterials requires further validation	High cost of nanomaterials and complex preparation processes increase the overall coating costs.
Structured Surface Coatings	Prevents biofouling through physical barriers and superhydrophobic properties.	Minimal impact on aquatic ecosystems, but susceptibility to wear could lead to failure.	High manufacturing and processing costs and relatively low maintenance costs.

## Data Availability

The original contributions presented in the study are included in the article, further inquiries can be directed to the corresponding authors.
